# The Impact of Obesity on Thyroid Autoimmunity and Dysfunction: A Systematic Review and Meta-Analysis

**DOI:** 10.3389/fimmu.2019.02349

**Published:** 2019-10-01

**Authors:** Rong-hua Song, Bin Wang, Qiu-ming Yao, Qian Li, Xi Jia, Jin-an Zhang

**Affiliations:** ^1^Department of Endocrinology & Rheumatology, Shanghai University of Medicine & Health Sciences Affiliated Zhoupu Hospital, Shanghai, China; ^2^Department of Endocrinology, Jinshan Hospital of Fudan University, Shanghai, China

**Keywords:** obesity, thyroid disease, thyroid autoimmunity, thyroid dysfunction, hypothyroidism, systematic review, meta-analysis

## Abstract

**Background:** To help inform decision making in the clinical setting, we carried out a systematic review and meta-analysis to estimate the association of thyroid disease risks with obesity.

**Methods:** Pubmed, Embase, Web of Science, Cochrane database and Google Scholar electronic databases were searched from inception to October 31, 2018 without language restrictions to explore the relationship between thyroid disorders and obesity. The relative risk (RR) or odds risk (OR) for thyroid disorders were pooled using the SPSS and STATA software.

**Results:** A total of 22 studies were included in the study. (1) Meta-analysis showed that obesity was significantly associated with an increased risk of hypothyroidism (RR = 1.86, 95% CI 1.63–2.11, *P* < 0.001). Meta-analyses after stratification further showed that obese population had increased risks of overt hypothyroidism (RR = 3.21, 95% CI 2.12–4.86, *P* < 0.001) and subclinical hypothyroidism (RR = 1.70, 95% CI 1.42–2.03, *P* < 0.001). (2) Further meta-analysis also showed obesity was clearly associated with Hashimoto's thyroiditis (RR = 1.91, 95% CI 1.10–3.32, *P* = 0.022), but not with Graves' disease. (3) In the meta-analysis of antibodies, obesity was correlated with positive thyroid peroxidase antibody (TPOAb) (RR = 1.93, 95% CI 1.31–2.85, *P* = 0.001), but not with positive thyroglobulin antibody (TGAb).

**Conclusions:** Obesity was significantly related to hypothyroidism, HT, and TPOAb, implying that prevention of obesity is crucial for thyroid disorders.

**Systematic Review Registration:** PROSPERO: CRD42018096897.

## Introduction

Since the rise of obesity epidemic worldwide, obesity has gained increasing attention and been regarded as a significant public health challenge globally for its wide-ranging adverse consequences on human health such as increased risks of diabetes (1), cardiovascular disease (2), and cancers (3).

The incidence of thyroid disorders, which mainly include thyroid dysfunctions and autoimmune thyroid diseases (AITDs), is increasing in these years. Thyroid dysfunctions include hyperthyroidism and hypothyroidism (4), both of which can be categorized into subclinical (only with changes in TSH) and overt stages (with changes in both TSH and thyroid hormones). AITDs, one of the most common autoimmune diseases, are characterized by autoantibodies against thyroid antigens, such as TSH receptor antibody (TRAb), thyroid peroxidase antibody (TPOAb), and thyroglobulin antibody (TGAb). They have two principal subtypes: Graves' disease (GD) and Hashimoto's thyroiditis (HT), which hold different clinical manifestations though have similar immunogenetic mechanisms (5). Patients with thyroid disorders also have a high risk of other non-thyroid diseases, such as cardiovascular diseases, cancer, obesity, and adverse pregnancy outcomes (6–9). Patients with thyroid dysfunctions or Graves' disease need long-term medical therapy or surveillance to optimize prognosis (10, 11). Identifying risk factors for thyroid disorders may help clinicians recognize individuals at risk for or with subclinical thyroid disorders and provide immediate treatment to improve patients' outcomes, and is crucial for elucidating the underlying pathophysiological mechanisms of these thyroid disorders.

Although previous studies have revealed that immune dysfunction, environmental elements and genetic factors all contribute to the pathogenesis of thyroid disorders, their pathology is not yet completely clear. It is well-known that obesity is associated with changes in hormones including thyroid-stimulating hormone (TSH) and thyroid hormones and is accompanied by several endocrine and metabolic diseases (12, 13). In clinic, it is well-known that hypothyroidism may induce obesity, so we propose a hypothesis that the relationship between obesity and thyroid disease may be bidirectional. Furthermore, if this relationship is bidirectional and if obesity indeed influences the risk of thyroid disorders, it is still incompletely elucidated how obesity influences the risk of thyroid dysfunctions and impacts the risk of thyroid autoimmunity. Although some studies have reported that obesity may be associated with dysfunctions of thyroid immunity and thyroid gland (14–16), these results are not entirely the same and even controversial. In addition, some of these studies have a relatively small sample size. Therefore, in this study, we conducted a systematic review and meta-analysis with aims to review the influence of obesity on thyroid diseases and uncover their association.

## Methods

### Registration

This systematic review and meta-analysis was conducted in accordance with the PRISMA guideline (17) and has been registered in the International Prospective Register of Systemic Reviews (PROSPERO, www.crd.york.ac.uk/PROSPERO, CRD42018096897).

### Literature Search

Pubmed, Embase, Web of Science, Cochrane database, and Google Scholar were searched from inception to October 31, 2018. The search in Pubmed used the following criteria: (obese OR obesity OR overweight) AND (thyroid autoimmunity OR Hashimoto's thyroiditis OR Graves' disease OR Graves hyperthyroidism OR hyperthyroidism OR hypothyroidism OR TPOAb OR TGAb OR thyroid peroxidase antibodies OR thyroid peroxidase antibody OR thyroglobulin antibodies OR thyroglobulin antibody OR thyroiditis). No restrictions were applied on language or publication period. Reference lists of eligible studies and reviews were also screened to identify more details.

### Eligibility Criteria

Inclusion criteria were as follows: (1) observational studies including cohort studies, cross-sectional studies and case-control studies; (2) studies comparing the risk of thyroid disorders of obese patients, who were defined as people with body mass index (BMI) ≥ 30 kg/m^2^ (in western population) or 28 kg/m^2^ (in eastern population), and normal controls, who were defined as people with 18.5 ≤ BMI < 24.9 kg/m^2^, or providing risk estimates for the associations of thyroid disorders with obesity; (3) studies analyzing thyroid disorders including hyperthyroidism, hypothyroidism, or AITDs; (4) studies providing risk estimates with 95% CI for the associations of thyroid disorders, such as relative risk (RR) and odds ratio (OR), or providing other data that could be transformed into risk estimates. Studies against any item of the eligibility criteria were excluded. Case reports and studies containing overlapping data were also excluded. Studies using overweight (24 ≤ BMI < 28 kg/m^2^), but not obesity (BMI ≥ 28 kg/m^2^) as the exposure were also excluded. The primary outcomes were the risk of thyroid autoimmunity, hypothyroidism or hyperthyroidism among obese patients and the secondary outcomes were the risk of AITDs, TPOAb positive, and TGAb positive among obese patients.

### Data Extraction

Data extraction was conducted using the extraction form, which mainly included study characteristics (the first author, publication year, design, country), participant characteristics (number, subgroups), outcomes (types of thyroid diseases), and adjusted matched factors.

### Quality Assessment

Quality assessment of included studies was conducted using Newcastle-Ottawa scale (NOS) based on participant selection (4 points), exposure evaluation (2 points), outcome evaluation, and confounders adjustment (3 points) (18). Studies scoring 5 or less were considered to have sub-optimal quality, and studies scoring 6 or higher were considered in good quality.

### Data Analysis

The pooled relative risk (RR) with 95% CI was used to evaluate the impact of obesity on the risk of thyroid autoimmunity and dysfunctions. To account for heterogeneity among included studies, data were pooled using random-effect meta-analysis (19). Heterogeneity was assessed using the *I*^2^ statistic, and *I*^2^ > 40% was considered high heterogeneity (20). Subgroup analysis was conducted based on types of thyroid autoimmunity, hypothyroidism and hyperthyroidism. Sensitivity analysis was conducted by excluding low-quality studies. Publication bias was assessed by the Begg's funnel plot and Egger's test (21). Trim-and-fill method was utilized when publication bias existed (22). All analyses were conducted in SPSS (version 25, IBMCorp) and STATA (version 12.0, StataCorp), and *P* < 0.05 was considered statistically significant.

## Results

### Search Results

**Figure 1:**
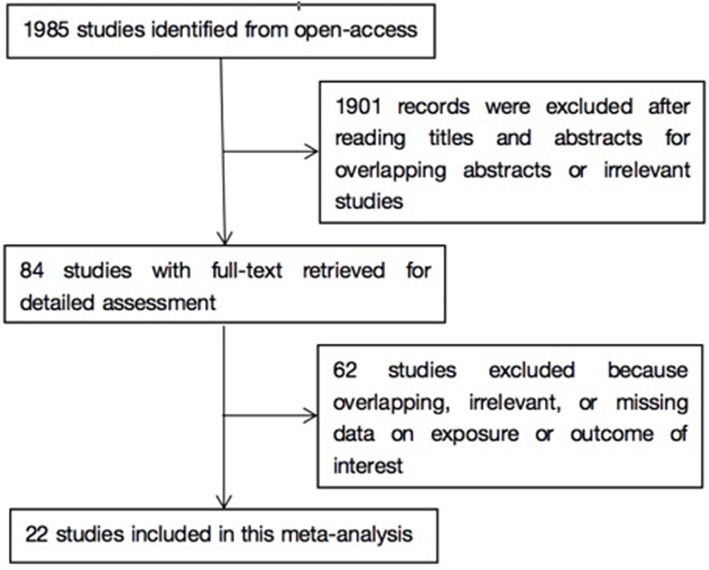
Flow chart of study selection in this meta-analysis.

As shown in Figure 1, literature search yielded 1985 related papers. After further careful abstracts viewing, 84 studies with full-text publications were retrieved for detailed assessment. After eliminating 62 papers with unrelated or ambiguous results, 22 papers were further analyzed in detail (14–16, 23–41). Table 1 lists the abstract items of the final 22 papers, including publication year, design, country or region, sample size, source of study sample, outcomes, adjusted matched factors, and quality assessment score.

**Table 1:** Characteristics of studies included in the meta-analysis.

**References**	**Design**	**Country**	**Participants**	**Subgroup**	**Outcomes (positive diagnostic criteria of antibodies)**	**Adjusted matched factors**	**Quality**
Rimm et al. (23)	Cross-sectional	USA	73,532 weight-conscious women	Adults	Hypothyroidism	None	6
Stichel et al. (24)	Case-control	Germany	290 obese and 280 healthy children	Children	SCH, TPOAb (>200 U/ml), TGAb (>100 U/ml)	Age	7
Knudsen et al. (25)	Cross-sectional	Denmark	4,082 eligible individuals after excluding subjects with previous or present overt thyroid dysfunction	Adults	SCH	Age, sex, region of inhabitancy, and tobacco smoking	8
Holm et al. (26)	Cohort	USA	115,109 women aged 25 to 42 at entry	Adults	GD	Age, duration of oral contraceptive use, age at menarche, parity, recent pregnancy, menopausal status, smoking status, alcohol intake, and physical activity level	9
Bhowmick et al. (14)	Case-control	USA	308 children with obesity and 286 non-obese children	Children	SCH	Age	8
Asvold et al. (27)	Cross-sectional	Norway	27,097 individuals older than 40 year of age who were without previously known thyroid disease	Adults	SCH, overt hypothyroidism	Age and smoking status	8
Gopinath et al. (28)	Cohort	Australia	951 participants without thyroid dysfunction	Adults	Hypothyroidism, overt hypothyroidism, SCH	Age and gender	9
Marzullo et al. (15)	Case-control	Italy	165 obese and 118 lean subjects	Adults	Hypothyroidism, overt hypothyroidism, SCH, HT, TPOAb (>35 IU/L), TGAb (>40 IU/L)	Age and gender	7
Dekelbab et al. (16)	Case-control	USA	191 obese and 125 non-obese children (younger than 18 years old)	Children	SCH	Age and gender	7
Somwaru et al. (29)	Cohort	USA	5,888 community-dwelling individuals aged 65 years and older	Adults	Hypothyroidism	Age, gender, race, education, and CHD at baseline	9
Hemminki et al. (30)	Cohort	Sweden	29,665 patients hospitalized for obesity and 367,459 individuals never hospitalized for obesity	Adults	GD, HT	Standardized incidence ratios (SIRs)	9
Ittermann et al. (33)	Cross-sectional	Germany	6,435 children (ages 3–10) and 5,918 adolescents (ages 11–17) from the “The German Health Interview and Examination Survey for Children and Adolescents” (KiGGS)	Children	Hyperthyroidism, hypothyroidism	Age, sex, smoking status, and environmental tobacco smoke	8
Ong et al. (31)	Cohort	UK	1,277 women and 1,185 men	Children	TPOAb (>100 IU/ml), hypothyroidism	None	9
Marwaha et al. (32)	Cross-sectional	India	13,691 children in the age group of 5–18 years	Children	SCH	None	8
Han et al. (34)	Cross-sectional	China	6,303 pregnant women	Adults	hypothyroidism, Overt hypothyroidism, TPOAb (>34 IU/ml), TGAb (>115 IU/ml)	Age, gestational weeks, TPOAb, TgAb, and UIC (stepwise manner)	8
Ghergherehchi and Hazhir (35)	Case-control	Iran	190 children who were overweight and obese and 133 children without obesity of the same age and sex were evaluated	Children	SCH	Age and sex	7
Korevaar et al. (36)	Cross-sectional	Netherlands	9,767 women during early pregnancy (≤18 week)	Adults	Overt hypothyroidism	Age, smoking, parity, ethnicity, and gestational age	8
Garcia-Garcia et al. (37)	Cross-sectional	Spain	1,317 healthy subjects aged 2–16 years	Children	SCH, HT	None	7
Amouzegar et al. (37)	Cohort	Iran	5,783 individuals were followed for 6 years	Adults	Overt hypothyroidism, SCH	Age, gender, smoking, waist circumference, and TPOAb	9
Valdes et al. (39)	Cross-sectional	Spain	3,928 individuals free of thyroid disease	Adults	SCH	Age, sex, smoking status, and UI concentrations	8
Ornaghi et al. (40)	Cross-sectional	Italy	309 pregnant patients	Adults	Hypothyroidism	Maternal age, parity, ethnicity, pre-gestational diabetes, use of antihypertensive medication before and during pregnancy, LDA prophylaxis, and GA at delivery	8
Wang et al. (42)	Cross-sectional	China	2,808 individuals	Adults	Hypothyroidism, SCH, HT, TPOAb (>34 IU/L), TGAb (>50 IU/L)	Age, gender, smoking, diabetes, uric acid, salt type and urinary iodine concentration	9

*SCH, subclinical hypothyroidism*.

### Obesity and Thyroid Dysfunctions

**Figure 2:**
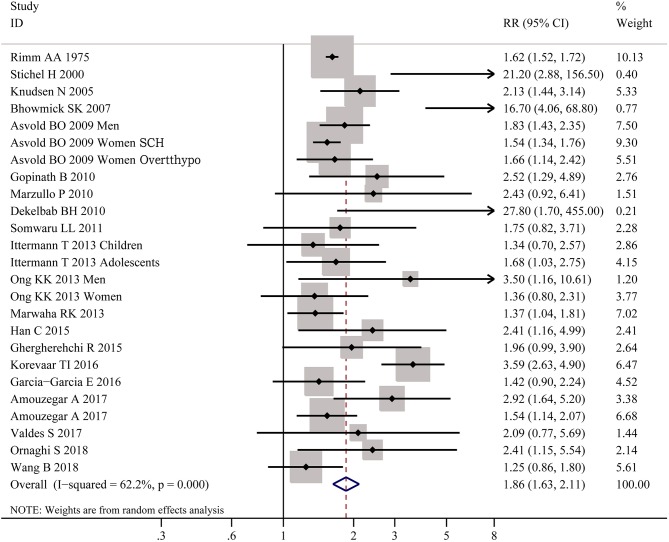
Forest plot for the risk of the whole hypothyroid disorders in obesity. SCH, subclinical hypothyroidism; Overtthypo, overt hypothyroidism.

**Figure 3:**
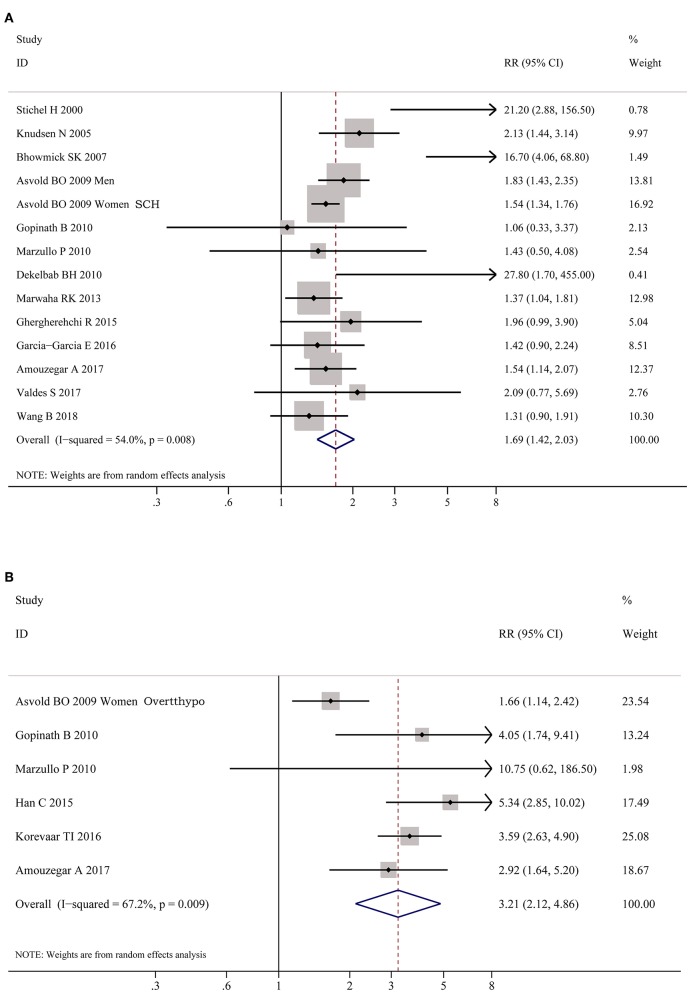
Forest plots for the risk of hypothyroid disorders in obesity. **(A)** Forest plot for the risk of overt hypothyroidism in obesity patients. **(B)** Forest plot for the risk of subclinical hypothyroidism in obesity patients. SCH, subclinical hypothyroidism; Overtthypo, overt hypothyroidism.

As shown in Figure 2, meta-analysis of the 22 studies indicated that obesity was significantly associated with the increased risk of hypothyroidism (OR = 1.86; 95% CI 1.63–2.11, *P* < 0.001). Further meta-analysis of 6 studies on hypothyroidism (shown in Figure 3) showed that patients with BMI ≥ 28 kg/m^2^ had an increased risk of overt hypothyroidism (OR = 3.21, 95% CI 2.12–4.86, *P* < 0.001). Likewise, meta-analysis of 14 studies on subclinical hypothyroidism (SCH) also showed that obese population had an 70% increased risk of subclinical hypothyroidism (OR = 1.70, 95% CI 1.42–2.03, *P* < 0.001). However, meta-analysis of studies on hyperthyroidism showed no significant association between obesity and an increased risk of hyperthyroidism (*P* > 0.05).

### Obesity and Thyroid Autoimmunity

**Figure 4:**
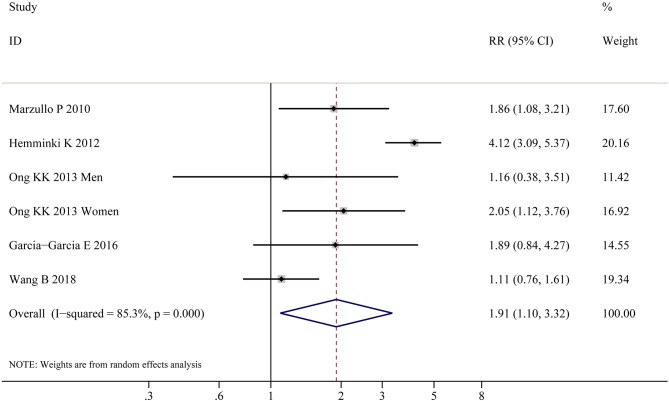
Meta-analysis of association between HT and obesity.

**Figure 5:**
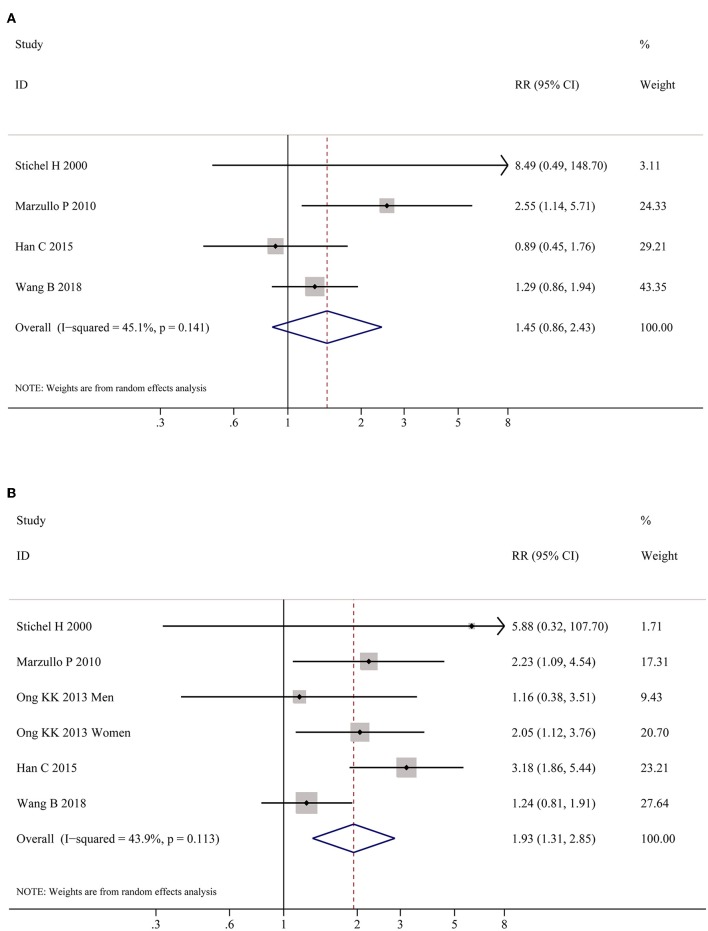
Meta-analysis of association between thyroid auto-antibodies and obesity. **(A)** Association between positive TGAb and obesity. **(B)** Association between positive TPOAb and obesity patients.

Table 2 shows the pooled estimates of AITDs risk in obese patients. Although obese patients had increased risk of AITDs, the difference was not statistically significant (*P* = 0.077). Similarly, meta-analysis of two studies on GD showed that obese population had no increased risk of GD (*P* = 0.852). But, there was a significant association between HT and obesity (OR = 1.91; 95% CI 1.10–3.32, *P* = 0.022), as shown in Figure 4. As shown in Table 2 and Figure 5, meta-analysis of thyroid antibodies (TGAb and TPOAb) revealed that there was a significant association between TPOAb positive and obesity (OR = 1.93; 95% CI 1.31–2.85, *P* = 0.001), but no such an association between TGAb positive and obesity. The risks of HT and TPOAb in obese population were increased by 91 and 93%, respectively.

**Table 2:** Meta-analysis of association of obesity with thyroid disorders.

**Analyses**	**No. of studies**	***I*^**2**^ (%)**	***P*-value**	**RR**	**95% CI**
AITDs	6	91.5	0.077	1.56	0.95–2.54
GD	2	90.4	0.852	0.94	0.51–1.75
HT	5	85.3	0.022	1.91	1.10–3.32
Hyperthyroidism	3	77.8	0.409	0.79	0.46–1.38
Hypothyroidism	20	62.2	0.000	1.86	1.63–2.11
Overt hypothyroidism	6	67.2	0.000	3.21	2.12–4.86
Subclinical hypothyroidism	14	54.0	0.000	1.70	1.42–2.03
TGAb	4	45.1	0.161	1.45	0.86–2.43
TPOAb	5	43.9	0.001	1.93	1.31–2.85

## Discussion

Obesity and thyroid disorders are two common conditions and there is an intriguing relationship between these two entities. Although available data have uncovered the relationship between thyroid disorder and body weight status, their results are inconsistent. For example, researchers have previously found that obese individuals have higher serum TSH levels (42, 43), while others have found no significant differences (44, 45). The aim of our study is to analyze these results systemically and also to reveal casual relationship between obesity and thyroid disorders.

A total of 22 researches with a size large enough were included in the present study. Clinically, it is easy to find that patients with hyperthyroidism often lose a lot of weight and regain it after remission. In contrast, patients with hypothyroidism often gain some weight and lose modest weight after thyroid hormone replacement. Therefore, it is a common sense that obesity is often regarded to be secondary to hypothyroidism (8). And the mechanisms by which hypothyroidism causes weight increase is supposed to be achieved via altered energy expenditure and appetite (41, 46).

Until recently, there have been more novel views identifying that thyroid disorders could well be secondary to obesity (47). Our meta-analysis showed that obesity was significantly associated with increased risks of hypothyroidism, including overt hypothyroidism and subclinical hypothyroidism, and could be accompanied by at least 1.86-fold increase of developing hypothyroidism. These results are concordant with other studies showing that lower levels of FT3 and FT4 or higher level of TSH are associated with high body weight (15, 25, 32, 48). The parthenogenesis of this relation is not yet entirely revealed, but some explanations have been proposed. Obesity is a chronic low-grade inflammation process; thus the cytokines and other inflammatory markers produced by over-loading adipose tissue, such as interleukin-1 (IL-1), IL-6, and tumor necrosis factor alpha (TNF-alpha), will be increased (46). These increased inflammatory cytokines may inhibit the mRNA expression of symporter sodium/iodide, then influence iodide uptake activity of human thyroid cells (49). These cytokines can also induce vasodilation and elevated permeability of blood vessels of thyroid gland, thus bringing morphological and functional changes in thyroid (46, 49). Leptin, a factor produced by adipocytes, also plays a role in chronic inflammation may result in the morphological changes in thyroid, and may also restrain the expressions of soidium/iodide symporter and thyroglobulin, thus inducing the changes of thyroid hormone levels in obese people (50). Some other studies found that this chronic inflammation status in obesity may also affect thyroid function by modulating the expression and activity of deiodinases (51, 52). The above researches may partially explain the mechanisms by which obesity may induce hypothyroidism (13, 49–53). Nevertheless, the etiology for the correlation of obesity and hypothyroidism still needs to be further elucidated in more in-depth studies.

Moreover, our meta-analysis also found the obese population had increased odds for thyroid autoimmunity. It is well-known that autoimmune diseases are caused by both genetic and immune pathogenesis. Our results are in accordance with previous reports showing that adiposity is a risk factor for many autoimmune inflammatory diseases, such as type 1 diabetes, multiple sclerosis, rheumatoid arthritis, and psoriatic arthritis (54–57). The mechanisms linking obesity and autoimmune disease are unclear. Some studies suggest that adipokines may play a vital role in immune disorders (58, 59). Adipokines, including leptin and interleukin-6, could mediate immune and inflammatory responses. Adipose tissue is crucial for maintaining normal immune function for humans (60, 61). Similarly, other observational researches also provide evidence that dysfunction in adipokines is associated with thyroid autoimmunity (62, 63). Meanwhile, meta-analysis of thyroid antibodies showed the correlation between TPOAb positive and obesity, and obesity is associated with a 93% increased risk of developing positive TPOAb. Leptin, which is mainly produced by adipocytes, is identified to mediate the immune system and contribute to increased production of TPOAb by shifting T helper balance toward to T helper 1 (Th1) cells phenotype and inhibiting the function of regulatory T (Treg) cells (64, 65). Autoimmune thyroiditis, mainly HT, is believed to be the main cause of hypothyroidism in iodine-sufficient regions, and thyroid auto-antibodies (TPOAb and TGAb) are the hallmarks of this disease (66). This may be another interpretation to explain the mechanism why obesity induces hypothyroidism.

Holm has reported that obesity may reduce the risk of hyperthyroidism (26). However, our meta-analysis including both Holm's study and another one showed no relationship between hyperthyroidism and GD with obesity. We speculate that this discrepancy is due to limited GD cases to reveal a fact in heterogeneous populations. In future, much larger and more ethnic researches are warranted.

In this study, we demonstrate the association between obesity and thyroid disorders, indicating that obesity may be a contributing factor for hypothyroidism, HT and positive TPOAb, and suggest that thyroid functions in obese population needs extra attention. So by synthesizing our present study and some other researches (13, 47, 49–53, 58–65), it seems reasonable to suggest that the relationship between obesity and thyroid disease is bidirectional; of course, it needs more studies to be elucidated. The present study still has some limitations. For instance, abnormal weight including overweight and underweight were barely explored. Additionally, most studies only explored the association between obesity and thyroid disorders, and barely investigated whether thyroid dysfunction is the cause or consequence of obesity, which needs further prospective cohort and causality studies to investigate.

In conclusion, obesity is significantly associated with hypothyroidism, HT and TPOAb, indicating that prevention of obesity is crucial for thyroid disorders.

## Author Contributions

BW designed the study and generated the hypotheses. BW and RS extracted the data. RS analyzed the data and wrote the first draft of the report, with support from QY, QL, and XJ. JZ and BW both revised the manuscript. All authors participated in interpreting the data and critically reviewed the paper.

### Conflict of Interest

The authors declare that the research was conducted in the absence of any commercial or financial relationships that could be construed as a potential conflict of interest. The reviewer RS and handling editor declared their shared affiliation.
